# Unlocking the mind: brain mapping for the exploratory quest of functional and cognitive networks in awake neurosurgery

**DOI:** 10.1007/s10143-026-04225-w

**Published:** 2026-04-21

**Authors:** Roberto Altieri, Ciro De Luca, Giovanni Maria De Martino, Assunta Virtuoso, Sergio Corvino, Giuseppe Pontillo, Oreste de Divitiis, Giuseppe La Rocca, Matteo Monticelli, Pietro Zeppa, Fabio Cofano, Antonio Melcarne, Carola Vera Junemann, Francesco Zenga, Daniela Pacella, Michele Papa, Diego Garbossa, Giovanni Cirillo, Manlio Barbarisi

**Affiliations:** 1 Multidisciplinary Department of Medical-Surgical and Dental Specialties, University of Campania “Luigi Vanvitelli”, Naples, 80131 Italy; 2 Laboratory of Neuronal Networks Morphology and Systems Biology, Department of Mental, Physical Health and Preventive Medicine, University of Campania “Luigi Vanvitelli”, Naples, 80138 Italy; 3 Department of Neuroscience and Reproductive and Odontostomatological Sciences, Neurosurgical Clinic, School of Medicine, University of Naples “Federico II”, Via Pansini, 5, Naples, 80131 Italy; 4 Department of Advanced Biomedical Sciences, University of Naples “Federico II”, Naples, Italy; 5 Institute of Neurosurgery, Fondazione Policlinico Universitario A. Gemelli IRCCS, Catholic University, Rome, 00168 Italy; 6 Dipartimento testa-collo, UOC Neurochirurgia, Azienda Ospedaliero-Universitaria di Parma, Parma, Italy; 7 Pediatric Neurosurgery Unit, Ospedale Infantile Regina Margherita, Azienda Ospedaliera Città della Salute e della Scienza Hospital, Turin, Italy; 8 Neurosurgery Unit, Department of Neuroscience “Rita Levi Montalcini”, University of Turin, Via Cherasco, 15, Turin, 10126 Italy; 9 Department of Public Health, University Federico II, Naples, Italy; 10 SYSBIO Centre of Systems Biology ISBE.ITALY, University of Milano-Bicocca, Milano, 20126 Italy

**Keywords:** Awake neurosurgery, Brain mapping, Neuronal networks, Systems biology, Brain lesions, Mentalizing, Cognition, Cluster analysis, Neuroanatomy

## Abstract

This retrospective study investigated the efficacy of intraoperative brain stimulation in 18 awake patients undergoing resection of intracranial lesions. The goal was to maximize resection while preserving cognitive function. We analyzed 91 stimulation sites using direct electrical stimulation (DES) during motor, language, and mentalizing tasks, with spatial response patterns identified via DBSCAN clustering. While all patients recovered their cognitive abilities, motor arrest was the most frequent response, highlighting the necessity of mapping motor pathways for preserving overall quality of life. Language responses confirmed classic functional hubs and the unpredictability of individual localization. Mentalizing, assessed via the “Reading the Mind in the Eyes” (RME) test, was minimally affected (1/91 sites). Clustered brain maps further support the necessity of DES to refine clinical nodes definitions, emphasizing the interplay of neural networks and subcortical connectivity in neuroplasticity. These exploratory findings suggest that comprehensive motor and language mapping during awake neurosurgery may support the stability of higher cognitive networks, though further research with larger cohorts and longitudinal neuropsychological tests is required to confirm these indirect preservation effects.

## Introduction

The traditional view of brain organization emphasized a rigid localization of functions, where specific areas were uniquely responsible for particular tasks [64]. This perspective suggested that damage to these areas would inevitably lead to permanent and irreversible functional deficits. A prime example is Broca’s area, long considered the exclusive center for speech production, with lesions invariably resulting in lasting language impairment [37].

However, increasing evidence challenges this rigid localizationist model. Studies, including those involving epilepsy surgery and tumor resections in awake patients, have demonstrated remarkable functional recovery even after damage to regions previously thought to be “eloquent” and indispensable. These observations suggest a more dynamic and distributed model of brain function, highlighting the brain’s capacity for reorganization and adaptation [16, 41, 52, 63].

The hodotopic model (derived from hodologic and topic) integrates the topographical arrangement of cortical functional nodes with the hodological organization of the large-scale white matter tracts that interconnect them [18]. This framework is supported by dynamic, distributed neural processes that operate across various temporal and spatial scales [33]. Such a model aligns with anatomical evidence in both healthy and clinical populations [11, 13], defining the connectome’s essential hubs that are vital for functional recovery [34]. Intensive research into primary brain functions has resulted in an increasingly precise characterization of this anatomy.

For instance, understanding the connections between traditional language areas and tracts such as the inferior fronto-occipital fasciculus (IFOF) [5], uncinate fasciculus [14], inferior longitudinal fasciculus (ILF) [5], and segments of the arcuate fasciculus [14, 39] is as critical as cortical mapping for predicting postoperative recovery. To illustrate, the extensive resection of the left temporal pole, involving the uncinate fasciculus and ILF, components of the indirect semantic ventral pathway, typically causes semantic deficits that resolve over months, provided the left IFOF (the direct semantic ventral pathway) remains intact [28, 29].

Moreover, when a tumor impairs such a network and causes preoperative semantic issues, surgical removal can actually improve linguistic performance [54]. Protecting the connectome is therefore vital for restoring function and enabling neuroplasticity to reweight pathways within the surviving network. This functional reorganization is supported by clinical data and increasingly by non-invasive tools like functional MRI [36]. Ultimately, the primary advancement in the field is not just identifying a few core structures but understanding the complex architecture of the connectome and its various subnetworks, including neural networks underlying mentalizing processes [22, 62].

Our study aim to contribute to the understanding of this functional connectome and the mechanisms of neural plasticity through awake craniotomy (AC), the gold standard surgical technique used to identify and preserve crucial brain functions during the excision of intrinsic brain lesions [16, 30, 53]. By integrating insights from intraoperative direct electrical stimulation (DES), real-time behavioral monitoring, and perioperative neuropsychological and neuroimaging data, we aim to reassess traditional models of cognition. This approach emphasizes the intricate interplay within and between neural circuits, leading to concepts of meta-networks and clusters and potentially highlighting the role of subcortical connectivity in neuroplasticity. Understanding these adaptable neural networks have profound implications for immediate improvements in patients’ quality of life (QoL) and advancements in fundamental neuroscience [19, 20, 61].

Previous research has shown that precise mapping of functional areas, particularly in adult low-grade glioma (LGG) cases, significantly improves long-term patient QoL [7, 23, 26, 27]. Specifically, our previous work showed that by halting resection upon the intraoperative identification of active functional nodes, it is possible to increase the extent of resection (EOR), exceeding 82% of tumor volume, while preserving a wide array of cognitive domains. While transient postoperative declines may occur, most functions, including linguistic expression, comprehension, and visuoperceptive abilities, typically return to baseline levels within 30 days of surgery [7]. This preservation of the cognitive scaffold is a primary driver of QoL, as it directly impacts a patient’s autonomy and social reintegration. In theory, expanding the mapping focus from purely motor and language skills to more complex cognitive functions, the surgical team should better balance aggressive oncological goals with the preservation of the patient’s long-term functional and social status. Numerous studies have utilized intraoperative probability maps generated through DES to identify and delineate motor and language functions [51, 52, 55]. As highlighted in a recent systematic review, there is a growing trend toward the use of standardized intraoperative language tests to ensure reproducibility across surgical centers [49]. In our cohort, language mapping was grounded in established tasks due to their high sensitivity in detecting bottleneck disruptions within the hubs of the dominant hemisphere.

However, investigations into cortical and subcortical responses related to emotion and cognition remain limited [7]. Recent work by Duffau et al. has highlighted the impact of surgery on QoL, specifically concerning cognitive, affective, and relational domains [38, 43, 63].

As we move toward mapping more complex cognitive domains like mentalizing, the field faces a challenge: while primary motor and language tasks are increasingly standardized [42, 48, 49, 59], higher-order tasks often lack a universally accepted intraoperative framework. This lack of standardization may contribute to the variability in negative findings across studies. Among various assessment tools, including functional MRI and DES, the “Reading the Mind in the Eyes” (RME) test has emerged recently as a promising, albeit still debated, intraoperative method for identifying brain areas subserving mentalizing functions [3, 17, 43, 56]. This study has two primary objectives: first, to explore the feasibility of intraoperative mentalizing assessments and their potential preservation through standard mapping protocols; and second, to descriptively map the spatial probability distribution of motor and language responses during DES.

## Materials and methods

### Study subjects

We retrospectively analyzed the surgical cases of patients treated in awake surgery. As a retrospective study, we acknowledge potential selection bias, as patients were selected based on their ability to tolerate awake surgery and perform complex tasks.

To ensure consistency and safety, patients were selected based on the following criteria:

Inclusion criteria:

1. Age between 17 and 75 years.
2. Neuroradiological diagnosis and subsequent anatomopathological confirmation of intrinsic brain lesion.
3. Lesion localization within functionally critical nodes, specifically the frontal (prefrontal gyrus), parietal (postcentral gyrus), insular lobes of both hemispheres, and left temporal lobes.

Exclusion criteria:

1. Lesions located far from the target functional networks, including the right temporal lobe, occipital lobes, and right frontal or parietal regions distal to the pre- and post-central gyri.
2. Technical impossibility of maintaining safe and definite tag marking for stimulation areas during resection.
3. Inability of the patient to perform neuropsychological tests or complex intraoperative tasks at baseline.

The sites of the lesions were selected based on their involvement in functionally critical regions where surgical injury traditionally carries a high risk of permanent neurological deficit. The focus on these specific regions was driven by their well-established roles in motor and language dominance. Conversely, the right temporal and occipital lobes were excluded as they typically represent non-eloquent zones for the primary motor and language functions targeted in this study. This selection allows for a high-density analysis of critical hubs where standard mapping (motor/language) and higher-order cognitive tasks (mentalizing) are most likely to converge or overlap, facilitating the exploration of our hypothesis.

Eighteen patients were included in the study, 9 males and 9 females with a mean age of 49 years. The patients were all right-handed. Eight patients (44%) had tumors located in the right hemisphere, and 10 (56%) in the left hemisphere. Right hemisphere lesions included: 1 fronto-temporo-insular (12.5%), 3 frontal (37.5%), 1 fronto-parietal (12.5%), 1 fronto-insular (12.5%), 1 parieto-insular (12.5%), and 1 parietal (12.5%). Left hemisphere lesions included: 3 frontal (30%), 1 parietal (10%), and 6 temporal (60%). Pathological diagnoses were: 9 glioblastomas (50%), 5 grade II oligodendrogliomas (IDH1 mutated, 1p/19q co-deleted, 27.7%), 2 grade II astrocytomas (IDH1 mutated, not 1p/19q co-deleted, 11%), 1 cavernoma (5.5%), and 1 dysembryoplastic neuroepithelial tumor (DNET, 5.5%). We acknowledge that these pathologies represent distinct biological entities with varying degrees of perilesional infiltration and neuroplastic potential. However, they were included to provide a comprehensive overview of the spatial responses encountered during our clinical awake mapping practice. The median preoperative and total tumor volume (TTV) on FLAIR images in LGGs and contrast-enhancing nodule plus FLAIR hyperintensity in glioblastoma was 30.05 cc (range: 10.8–201.6 cc). The median postoperative TTV was 3.5 cc (range: 0–38.05 cc). These characteristics are summarized in Table 1.

**Table 1:** Distributions of the population characteristics

	Gender	9 Male / 9 Female
Demographics	Mean Age	49 years
	Handedness	100% Right-handed
Lesion Laterality	Left Hemisphere	10 (56%)
	Right Hemisphere	8 (44%)
Pathology	Glioblastoma	9 (50%)
	Grade II Oligodendroglioma	5 (27.7%)
	Grade II Astrocytoma	2 (11%)
	Cavernoma	1 (5.5%)
	DNET	1 (5.5%)
Tumor Volume (TTV)	Median Preoperative	30.05 cc (Range: 10.8–201.6)
	Median Postoperative	3.5 cc (Range: 0–38.05)

### Intraoperative protocol

All patients followed a standardized intraoperative protocol. Awake surgery and brain stimulation followed standard surgical procedures without modifications for research purposes. Dexmedetomidine (0.7–2.0 µg/kg/hour) was administered for sedation during the initial surgical phases. Sedation was discontinued following craniotomy and prior to dural incision. The craniotomy exposed the tumor and 2–3 cm of surrounding cortical tissue. Tumor resection was guided by neuronavigation and 5-ALA in cases of glioblastoma (GBM). Bipolar DES was performed using electrodes with a 5 mm inter-electrode distance. A classic Ojemann paradigm (60 Hz, biphasic, 0.5–1 msec pulse width) was employed. Stimulation-induced seizures were managed by irrigation of the exposed brain surface with cold saline. In cases refractory to irrigation, intravenous levetiracetam (1 g) was administered. Brain Mapping (BM) commenced with an initial stimulation intensity of 2.5 mA, progressively increased up to 10 mA, or until identification of the negative motor network (NMN) around the inferior frontal gyrus (IFG) and sensorimotor cortex. The stimulation intensity was recorded for each mapping procedure. To ensure technical consistency, all BM procedures were performed by a single experienced surgeon (R.A.). The NMN was identified using a dual-task paradigm consisting of counting (to monitor for speech arrest) and continuous, rhythmic contralateral upper limb movements (specifically continuous rhythmic flexion-extension of the forearm). While counting is an automated task, it provided a stable baseline to identify motor/speech interference before proceeding to higher-level lexico-semantic tests.

Total motor arrest (TMA) during a dual task prompted further mapping at the same stimulation amplitude. To identify the functional nodes within the surgical field, the following standardized intraoperative tasks were utilized:

1. DO80 (object naming): to evaluate lexical retrieval and speech production. A positive feedback was defined as an immediate speech arrest, anomia, or paraphasia during stimulation, provided that spontaneous speech remained intact.
2. Pyramid and Palm Tree (PPT) test: to assess semantic association and the ventral semantic pathway. Patients are asked to match a target image with one of two choices based on a semantic relationship. Disruption (positive feedback) was defined as semantic paraphasia or the inability to establish a logical link between images.
3. Reading the Mind in the Eyes (RME): to target the social brain and affective Theory of Mind (ToM). Patients viewed printed images of the eye region and must choose the correct mental state from four options. Participants are asked to select the mental state that best describes what the person in the photograph is feeling. Positive feedback was recorded if the patient exhibited an incorrect choice (or latency > 5 s) during stimulation. The ability to attribute independent mental states to oneself and others is defined as mentalizing.

Three consecutive positive feedbacks following DES were defined as a positive response, adhering to the widely accepted surgical paradigm for functional mapping to minimize the risk of false positives and ensure the reliability of the identified eloquent site [16, 55]. We acknowledge that this battery may not capture the full complexity of executive functions, but it served as a feasible intraoperative compromise for maintaining the onco-functional balance.

The RME test was selected because in non-surgical settings it is considered ameasure of the affective ToM [10, 47, 50, 57], which relies on a distributed network including the inferior frontal gyrus, the posterior temporal-parietal junction, and the insula, frequently involved in the gliomas included in this study [15]. Unlike simpler tasks, the RME test requires the integration of subtle social cues and mental state attribution, making it theoretically able to detect subtle cognitive shifts that standard language or motor mapping might overlook. We acknowledge several limitations regarding the RME test in the intraoperative setting: it requires significant cognitive load and visual processing time. The surgical environment reduces the sensitivity of the assessment compared to full bedside testing. Moreover, while the RME test is sensitive to the mentalizing network, it also involves visual and linguistic components (decoding and labeling). Therefore, a positive response (failure in the task) must be carefully cross-referenced with motor and language mapping to ensure the deficit is specifically related to mentalizing [43, 63]. Positive sites were marked with sterile tags and documented by digital photography using the operative microscope before and after tumor resection. DES responses were also recorded in a dedicated database.

### Preoperative, and post-operative protocols

Preoperative (baseline) and postoperative (5 days and 30 days) neuropsychological assessments were conducted by trained speech therapists. NOMS (National Outcomes Measurement System) scale was used as a therapist-rated, ordinal functional scale (ranging from 1 to 7) for the severity of impairment in communication and cognitive functions in various domains, including speech and language (expression, comprehension, reading, writing), cognition (attention, memory, problem-solving and pragmatics), and visuospatial orientation, as previously described [6, 7, 41].

Postoperative MRI was performed within 48 h to assess the extent of resection using the Berger formula. Tumor volumes were calculated via manual segmentation using Horos software for macOS by two neurosurgeons with expertise in neuro-oncological surgery and neuroradiological imaging. Preoperative and postoperative MRI scans were used to calculate the enhancing nodule (EN) volume, FLAIR volume, TTV. The extent of resection for ENs and TTV was then determined.

### Neuroanatomical navigation and 3D mapping

**Fig. 1:**
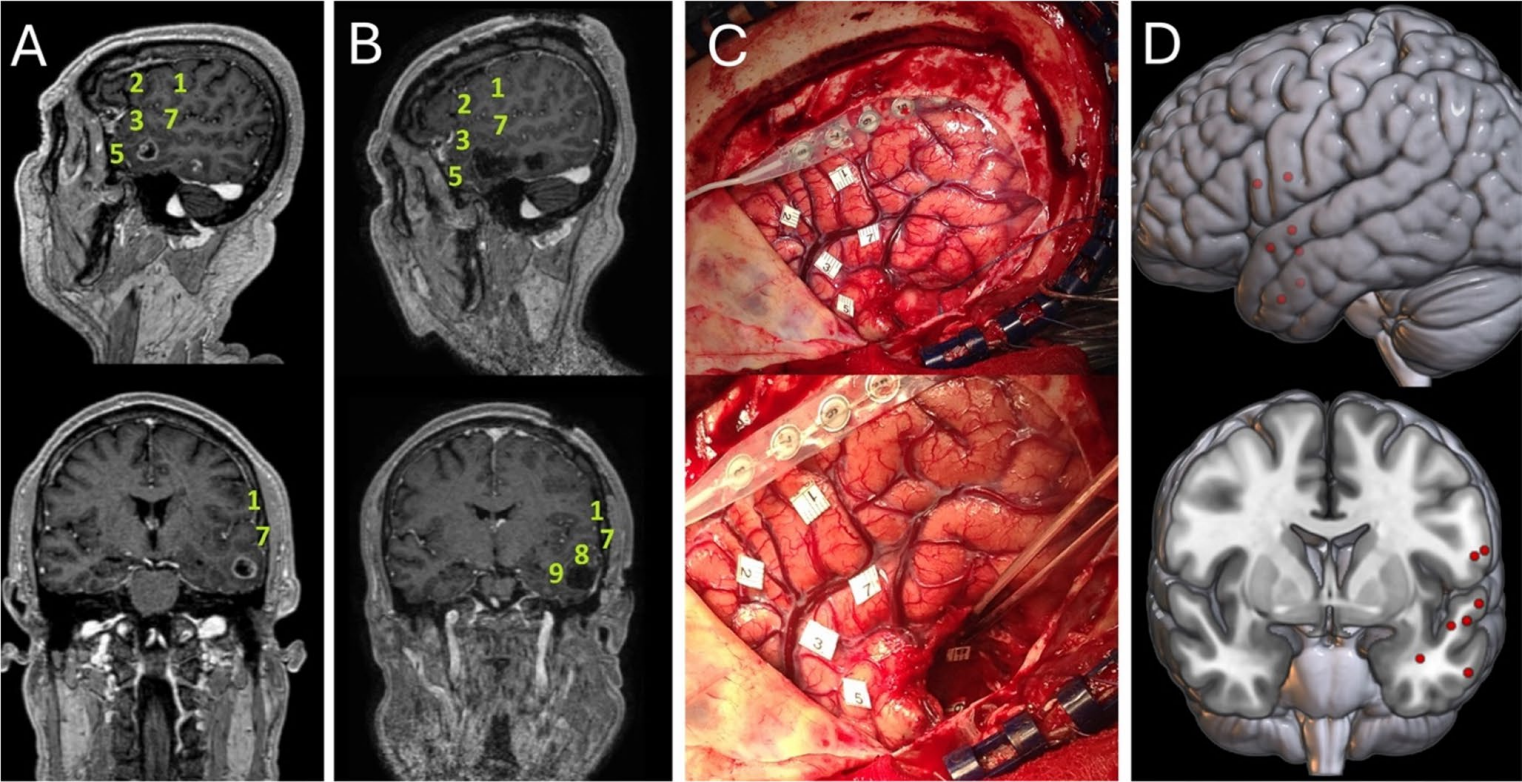
Preoperative (**A**) and postoperative (**B**) MRI scans of a patient affected by left temporal glioblastoma (GBM). Numbers on the cortical and subcortical surfaces were positioned by comparing the MRI with an intraoperative picture of the labeled brain after mapping (**C**). In (**D**), these points are transposed onto the 3D Montreal Neurological Institute (MNI) brain template

Intraoperative photographs were compared with preoperative and immediate postoperative MRI scans to identify tag positions. These positions were then transposed onto the 3D Montreal Neurological Institute (MNI) brain template, assigning x (right-left), y (posterior-anterior), and z (inferior-superior) coordinates using MRIcroGL (https://www.nitrc.org/projects/mricrogl). Stimulation sites were identified intraoperatively relative to anatomical landmarks (sulci, gyri, and distance from the tumor margin). These coordinates were transposed to the MNI template by two independent authors (blinded to each other’s assignments) using a standardized anatomical atlas (a neurosurgeon and a neuroradiologist). A third author was assigned to review the location and validate it or to settle discrepancies (neuroanatomy professor). We estimate an inherent spatial error (3–5 mm) associated with this manual approach (Fig. 1).

### Statistical analysis

Data are summarized as mean and standard deviation (SD) or median and interquartile range (IQR) for continuous variables and as absolute frequency and percentages for categorical variables. Spatial distribution of the stimulation sites was reported using coordinates x (right to left), y (posterior to anterior) and z (inferior to superior). To investigate the association between the stimulation sites and the responses, spatial cluster analysis was implemented using the three coordinate values. To identify the response regions, considering their irregular and concave shapes, we used the Density-Based Spatial Clustering of Application with Noise (DBSCAN) algorithm. We acknowledge that a manual MNI registration can lead to an inherent spatial error (approximately 3–5 mm) The parameters ε (epsilon) and MinPts were determined through visual inspection of 3D plots and comparison of the Bayesian Information Criterion (BIC), in other words, the parameters were determined through a data-driven approach to ensure reproducibility. To ensure the stability of the clusters, we performed a sensitivity analysis by varying epsilon in increments of 0.5 mm and comparing the resulting cluster configurations using the BIC. Assuming for the present data a negligible inter-individual variability, the DBSCAN clustering analysis was performed considering the 91 individual stimulation sites as independent data points. Differences between clusters were assessed using ANOVA for continuous variables or Fisher’s exact test for categorical variables. Normality of spatial coordinates and other data was assessed through the Shapiro-Wilk Test. Non-parametric tests were employed to assess changes in NOMS performance at three timepoints (baseline preoperatively, 5 days postoperatively, and 30 days postoperatively). The performance scoring system admits integers from 1 (totally impaired) to 7 (no impairment) with 20% impairment gap between each group. The Kruskal-Wallis test was utilized to compare the median values of each cognitive domain across these time points, followed by post-hoc Dunn’s test with Bonferroni correction for multiple comparisons.

## Results

### Intraoperative findings

A total of 91 functional sites were identified through intraoperative direct electrical stimulation (DES), comprising 69 cortical (75.8%) and 22 subcortical (24.2%) responses. The mapping revealed three key observations: first, total motor arrest (TMA) was the most frequent functional disruption across both hemispheres; second, language-related responses were highly concentrated in the dominant left temporal and parietal lobes; and third, the mentalizing network (RME test) demonstrated remarkable resilience, with only a 1.1% failure rate. These findings may suggest that while motor and language nodes are overall considered as main hubs, densely packed and easily disrupted, higher-order cognitive networks could be more distributed or redundant.

Cortical responses were distributed between the left (46.1%) and right (29.6%) hemispheres. While TMA was the predominant response, the data highlighted that identical anatomical sites could elicit diverse behaviors. For instance, stimulation of the right triangular part of the inferior frontal gyrus (IFGtriang) produced both TMA and paresthesia.

Within the superior frontal gyrus (SFG), TMA was induced once on the dorsolateral left side and twice on the right. Similarly, the right middle frontal gyrus (MFG) yielded two TMA responses, while one was obtained by stimulating the right supplementary motor area (SMA). Further mapping revealed two TMA responses in the left opercular part of the inferior frontal gyrus (IFGoperc) and one in the left paracentral lobule (PCL). The left postcentral gyrus (PoCG) was predominantly associated with motor and language disruptions (9 TMA, 1 anomia, 2 limb arrests), while the right PoCG resulted in four TMA. These responses, occurring also outside traditionally defined functional clinical nodes, underscore the intricate networking of the system. The right precentral gyrus (PreCG) proved highly active with 14 responses, primarily TMA alongside dysarthria and speech arrest. Also the right Rolandic operculum (ROL) yielded 2 TMA.

Regarding language-specific hubs, the majority of events were concentrated in the left temporal and parietal lobes. The left middle temporal gyrus (MTG) yielded two cases of anomia and two of semantic paraphasia, while the superior temporal gyrus (STG) was associated with two anomia responses, including one at the temporal pole (TPOsup). The left supramarginal gyrus (SMG) also appeared to be an essential hub of the connectome, involving four cases of anomia and one of phonemic paraphasia. Stimulation of the insula resulted in one instance of phonemic paraphasia, and the left ROL was involved in eight responses (6 TMA, 1 speech arrest, 1 anomia/phonemic paraphasia).

**Fig. 2:**
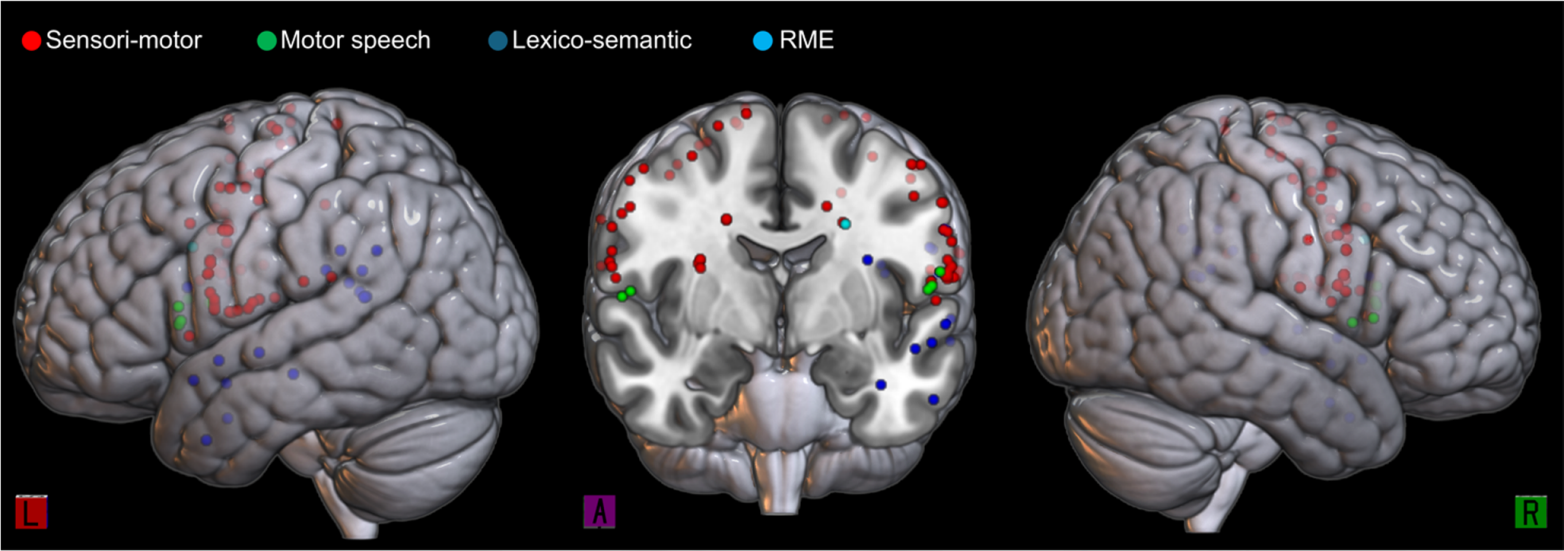
Spatial mapping of all responses on the MNI model. Among the 91 identified cortical and subcortical sites, only one positive RME response was detected (light blue dot) on the left SLF

Subcortical stimulation yielded 22 total responses, with 60% in the right hemisphere and 40% in the left. Purely motor responses were localized to the corona radiata (CR), where stimulation produced perioral, lower limb, and hemisomal contractions. TMA was also elicited from the right fronto-striatal tract (FST). In the dominant hemisphere, language-related disruptions were prominent within the white matter: the left IFOF evoked two cases of semantic paraphasia, the ILF produced one case of anomia, and the middle longitudinal fascicle (MLF) resulted in three language-related events (2 anomia, 1 phonemic paraphasia). Notably, higher-order cognitive interference was extremely rare; only one site (1.1%), localized to the right superior longitudinal fasciculus (SLF), resulted in a failed RME test. This finding highlights the significant resilience of the mentalizing network during subcortical mapping compared to the higher frequency of disruptions observed in motor, sensory, and language tracts (Fig. 2).

### Clustering analysis

**Fig. 3:**
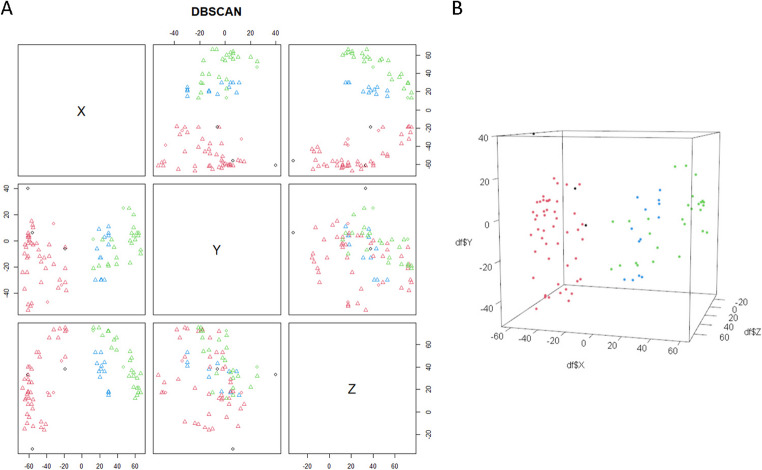
**A**) Projections by dimension of the DBSCAN clusters based on spatial coordinates. Cluster 1 (in red) clinically characterized by motor arrest and language abnormalities in the dominant hemisphere;, Cluster 2 (in green) expresses the non-dominant motor arrest network; and Cluster 3 (in blue) outlines the mapping of positive motor activation. The outliers are in black. **B**) 3D visualization of the same DBSCAN clusters

Spatial cluster analysis, utilizing the DBSCAN algorithm, successfully identified three distinct functional hubs for motor and language responses, providing a preliminary descriptive roadmap. While these high-density zones identify frequent functional disruptions within this specific group, they should be viewed as exploratory findings rather than a definitive predictive tool for all neurosurgical populations, given the small and heterogeneous nature of the cohor. t. Out of 91 observations, 48 were grouped into Cluster 1, 27 into Cluster 2, and 13 into Cluster 3, while only 3 points were classified as outliers (Table 2; Fig. 3).

The clinical significance of these clusters lies in their ability to predict functional risk during resection. Cluster 1, characterized by its posterior and left-lateralized spatial distribution, represents a critical bottleneck where motor execution and language production pathways converge. The high association with both motor arrest (50%) and language disruption (46%) indicates that surgical violation in this zone carries a high risk of multi-domain deficits, necessitating the most conservative resection margins.

In contrast, Cluster 2 encompasses the right-lateralized and most anterior stimulation sites and was predominantly associated with motor arrest (85%). Clinically, this identifies the essential nodes of the non-dominant premotor and motor cortex, where stimulation typically results in a total cessation of movement rather than specific muscle contractions.

Finally, Cluster 3 is located in the ventral aspect of the mapped region and exhibited a significant association with contraction responses (69%). For the neurosurgeon, this cluster identifies the deeper, descending corticospinal fibers and sensory pathways. Unlike the cortical arrest zones, proximity to this ventral cluster is signaled by involuntary motor activity, providing a distinct functional boundary during subcortical dissection. By identifying these high-density zones, the DBSCAN analysis suggests a path from descriptive mapping toward a a potentially predictive tool, allowing through further standardization to anticipate the specific type of functional disruption likely to be encountered based on the anatomical trajectory of the resection.

**Table 2:** Distributions of the responses and of the coordinates among clusters. Data are reported as mean (SD) or as frequency (percentage)

Variable	Cluster 1 *N* = 48	Cluster 2 *N* = 27	Cluster 3 *N* = 13	*p*-value
Response				**< 0.001**
Motor arrest	24 (50%)	23 (85%)	1 (7.7%)	
Contraction	2 (4.2%)	0 (0%)	9 (69%)	
Language	22 (46%)	2 (7.4%)	1 (7.7%)	
X	-49 (16)	47 (17)	23 (5)	**< 0.001**
Y	-14 (19)	-1 (13)	-9 (16)	**0.004**
Z	26 (27)	44 (21)	34 (13)	**0.010**

### Clinical results

**Fig. 4:**
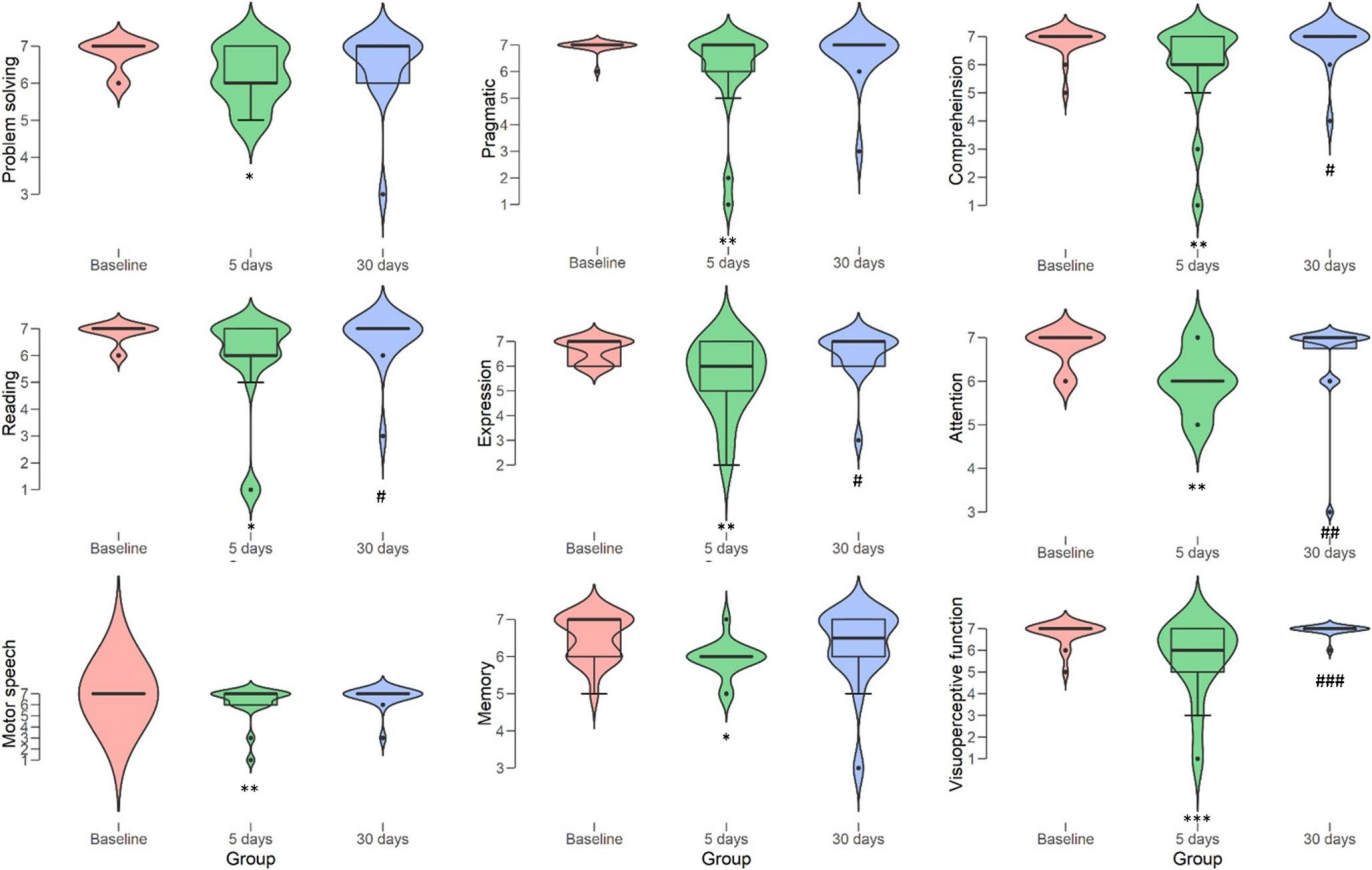
Distribution plots of all the affected clinical performances using violin and boxplot data representation. Statistical significance is indicated as *p < 0.05, **p < 0.01, ***p < 0.001 versus baseline and #*p* < 0.05, ##*p* < 0.01, ###*p* < 0.001 between postoperative groups

The median EOR for TTV was 83.50% (range: 12.50–100%). Complete EN resection (100% EOR) was achieved in all GBM cases. Patients did not exhibit postoperative deterioration in writing functions, consistent with our previous report [7]. Notably, we registered median cognitive scores returned to baseline levels at 30 days, although increased inter-individual variability suggests that a full restoration of the baseline distribution was not achieved for all patients. All the investigated cognitive fields (comprehension, motor speech, expression, reading, pragmatics, attention, problem-solving, memory, and visuoperceptive function), were modestly impaired 5 days after surgery. The data and the statistical comparison between different groups are plotted in Table 3; Fig. 4, allowing to observe also the increased dispersion of IQR.

**Table 3:** Comparison of the performances at baseline (preoperatively) and after 5 or 30 days after surgery for each investigated domain. Data are reported as median (IQR)

	Baseline	5 days	30 days	Kruskal Wallis	Dunn-Bonferroni (*p* value)		
	Median (IQR)			*p* value	Baseline vs. 5 days	Baseline vs. 30 days	5 days vs. 30 days
Comprehension	7 (0)	6 (1)	7 (0)	**0.009**	**0.009**	1	**0.016**
Expression	7 (0)	6 (2)	7 (1)	**0.007**	**0.008**	1	**0.013**
Reading	7 (0)	6 (1)	7 (0)	**0.027**	**0.021**	1	**0.046**
Writing	7 (0)	7 (1)	7 (0.25)	0.126			
Pragmatic	7 (0)	7 (1)	7 (0)	**0.012**	**0.005**	0.544	0.181
Motor speech	7 (0)	7 (1)	7 (0)	**0.006**	**0.003**	0.506	0.054
Attention	7 (0)	6 (0)	7 (0.25)	**0.002**	**0.001**	1	**0.005**
Memory	7 (1)	6 (0)	6,5 (1)	**0.027**	**0.015**	0.888	0.296
Problem-solving	7 (0)	6 (1)	7 (1)	**0.049**	**0.025**	0.78	0.128
Visuoperceptive function	7 (0)	6 (2)	7 (0)	**< 0.001**	**0.006**	0.74	**< 0.001**

## Discussion

Maximal resection of brain tumors significantly increases overall survival especially in LGG [45]. However, the safety of maximal resection and the postoperative QoL needs to be balanced to avoid unbearable postoperative morbidity. Brain stimulation in the awake patient is essential, guiding the neurosurgeon to take intraoperative decisions and mapping the boundaries of safe resection. Still, it can also unravel neural networks, redefining the rigid scheme of the classical eloquent areas.

Preoperative and postoperative neuropsychological assessment is essential in understanding the impact on patient cognition of safe resection [23, 27, 38]. As a clinical hypothesis, the avoidance of motor or sensory loss needs to be considered together with cognitive and mentalizing integrity. Although our current data provides only minimal empirical evidence for a specific mentalizing hub, the conceptual framework of preserving higher-order social cognition remains a goal of modern functional-safe neurosurgery. We analyzed retrospectively how monitoring intraoperative tasks could affect patient outcomes and performances in multiple domains of cognition. In principle, using intraoperative tasks furnishes a more reliable prediction of postoperative autonomies in daily life, compared with preoperative evaluation. Moreover, directly stimulating cortical and subcortical areas allows for a patient-tailored approach and generalized brain mapping. The stimulation of neural networks through DES combined with intraoperative dual task, DO80, PPT, and RME tests permitted the exploration of motor, sensory, phonological, verbal semantic processing, mentalizing, speech, and language.

Utilizing specific intraoperative tasks, we accurately checked cognitive and sensorimotor functions in different locations, some of which were unexpected. The TMA was the most registered behavior with 46 responses out of 91. As anticipated, stimulating the primary motor cortex in the PreCG prompted the highest number of TMA, but less than 40% of the total. The analysis revealed that the most strongly associated cluster for motor arrest responses (85%) is Cluster 2, characterized by stimulation sites located on the right hemisphere anteriorly in the SFG and MFG and posteriorly in the PoCG. Sparing neural networks for redirecting motor behavior is crucial for the system’s plasticity after the excision of a brain lesion. Our data support the validity of dual task paired with DES to clearly define the area of response, especially in the right hemisphere.

All the study subjects were right-handed and none of them manifested language-related behavior in the right hemisphere. Indeed, Cluster 1, characterized by the left-lateralization and posterior distribution of the responses, combines motor arrest and language responses in the sensorimotor cortex of PoCG and ROL, with pure language responses in the SMG, Insula, and temporal lobe (STG, MTG, and TPOsup) or the subcortical IFOF. Registering this cluster of responses underscores the potential importance of certain functional nodes for clinical outcomes. Moreover, Cluster 1 reveals that language, combined with motor arrest, can be triggered by the stimulation of unpredictable localizations.

The positive motor behavior clustered as the most ventral of the registered positions (Cluster 3), and was evoked mainly by stimulating the CR of both hemispheres. The difference in motor behavior is remarkable, as cortical stimulation prompted mostly motor arrest responses while subcortical stimulation of white matter tracts elicited 12 out of 14 positive motor responses.

Mentalizing, on the other hand, was not altered by the carried stimulation, except in one site, located in the right SLF of a patient with right parietal oligodendroglioma.

In the pursuit of optimizing the surgical protocols and onco-functional balance, it is mandatory to find a reliable protocol, preserving the cognitive abilities in patients with intraparenchymal lesions but without prolonging the surgery or carrying non-essential tests during awake surgery. The RME test has been proposed as an intraoperative diagnostic tool to identify neural networks associated with higher cognitive functions and mentalizing during DES in awake patients [1, 55, 58].

In our report, we determined just 1 out of 91 points of stimulation interfered with the RME test. Nonetheless, the neuropsychological assessment confirmed that no permanent deficits in common cognitive domains were developed. In our real-life experience, the identification and preservation of cortical and subcortical areas through the dual task, theDO80 and PPT tests have demonstrated to accomplish the desired outcome [1, 6, 44].

Moreover, eliciting responses with the RME test proved challenging, contrasting with the reliable elicitation of motor and language-related responses. The surgery protocols even without the RME test can provide safety and maximal tumor excision measured both in terms of postoperative cognitive domain preservation and EOR [32].

Our evidence suggests that a simplified, time-sparing monitoring protocol focused on primary motor and language hubs may contribute to preserving the overall postoperative QoL. We hypothesize that by ensuring the integrity of these essential bottleneck areas, the resilience of interconnected higher-order networks might be indirectly maintained, even in the absence of specific intraoperative cognitive testing [2, 24, 25, 34]. Furthermore, the expansive growth pattern of high-grade tumors may primarily displace rather than extensively infiltrate white matter tracts, preserving their functional integrity despite gross anatomical alterations [4, 8, 9].

However, these findings must be interpreted with caution. While our patients demonstrated stability in the postoperative period, the relationship between primary mapping and higher-order preservation remains a hypothesis requiring further investigation. Our results should be viewed as supporting the feasibility of motor and language mapping approach in standard awake neurosurgery, rather than providing definitive evidence that higher cognitive mapping is redundant [7, 12, 35, 60].

We established a clustered map of brain areas confirming the need for DES in awake surgery to overcome the intrinsic limitation of the classical localization for eloquent areas. Our data further supports the intricate interplay within the systems biology of neural circuits organized in meta-networks and clusters with a pivotal role of subcortical connectivity.

The established link between motor dysfunctions and cognitive impairment, coupled with the neurotrophic effects of physical exercise on the hippocampus and cerebral cortex, underscores the intertwined motor and cognitive domains, particularly visuoperceptive and executive functions [21, 40]. Additionally, preserving language domains is crucial due to the multiple cognitive functions associated with the white matter tracts such as the IFOF and the arcuate fasciculus [31, 46]. The observation that only 1.1% of stimulation sites disrupted the RME task warrants a critical appraisal. On one hand, this low rate of interference may suggest a high degree of network resilience. However, we must also consider the methodological limitations of the RME task in the intraoperative setting. The task primarily involves a decoding process (reading emotions from static images), which may not capture the dynamic, reasoning components of mentalizing that are more susceptible to surgical manipulation. Furthermore, insufficient sensitivity could stem from the adaptation of the test for the operating room. Therefore, while our results are encouraging for the preservation of gross mentalizing abilities, they do not preclude the existence of subtle postoperative social-cognitive deficits that might only be detectable through more granular, long-term neuropsychological assessments.

We must acknowledge several limitations to our study, most notably the small and heterogeneous patient cohort. With only 18 patients, the diversity in tumor types, ranging from slow-growing low-grade gliomas to aggressive glioblastomas, one DNET, and a cavernoma, introduces variables in neuroplasticity and white matter displacement that may affect mapping results. Drawing generalizable conclusions or definitive clusters from such a limited dataset requires extreme caution. While the analysis of 91 independent stimulation sites allows for a detailed descriptive spatial map, these findings should be considered exploratory. The clustering patterns identified via the DBSCAN algorithm represent high-probability zones within this specific group, but they cannot yet be generalized as universal landmarks for cognitive networks. Larger, multi-center studies with more homogeneous cohorts are required to validate these predictive models.

Furthermore, the RME test may lack the sensitivity for intraoperative use, explaining the low interference rate; this does not imply that higher cognitive functions are not vulnerable, but highlights the need, eventually, for more sensitive intraoperative tools. The study does not select or match demographic and tumor characteristics, potential selection bias and confounding variables, such as volume of the lesion and duration, cannot be entirely excluded due to the study’s retrospective design. The inclusion of a lesion like cavernomas alongside gliomas and DNET introduces further heterogeneity. While all types of lesions require precise mapping, through mechanical dislocation of the surrounding parenchyma, the lack of tumor-related neuroplastic effects (e.g., excitotoxicity) in vascular lesions may mean that the functional reorganization observed in this subgroup differs from that of the glioma or DNET subgroups. To enhance the analytical power of anatomical clusters, functionally distinct cognitive functions were grouped into broader categories, which may have obscured nuanced effects of intraoperative stimulation.

The pre- and postoperative neuropsychological tests were not specifically designed to highlight mentalizing processes. While NOMS testing, rated by trained speech therapists, provides a robust measure of daily functional independence, it is not a neuropsychological test based on population-normative data. Consequently, the results reflect gross functional outcomes rather than subtle cognitive fluctuations. Finally, the definition of 5 days and 30 days postoperative neuropsychological deficit, based on a simple comparison to preoperative baseline, may be less stringent than criteria employed by other researchers, potentially impacting the comparability of results [38, 45, 51]. Hence, we must differentiate our hypotheses from a definitive proof of total cognitive preservation. The current data do not definitively demonstrate that higher functions are preserved in their entirety, but rather that they are not acutely disrupted by focal stimulation in the tested areas. Future studies utilizing more sensitive, dynamic social-cognitive tasks and longitudinal neuropsychological follow-up are required to confirm whether this indirect preservation hypothesis holds true across the full spectrum of postoperative recovery. Consequently, the exploratory nature of this study necessitates cautious interpretation, and validation through prospective studies with larger datasets is warranted.

The integration of advanced neuroimaging techniques, such as functional MRI (fMRI) and diffusion tensor imaging (DTI), could enhance intraoperative mapping accuracy by providing detailed representations of cognitive and metacognitive neural networks. These technologies, combined with intraoperative stimulation mapping, may refine surgical strategies and improve patient outcomes.

## Conclusions

Exploring the dynamic nature of neural networks provides crucial insights into systems biology, with significant benefits for both patient well-being and basic neuroscience. Despite the proposal of the RME test for intraoperative cognitive mapping, its practical application presents challenges. Our study utilized a data-driven spatial clustering approach to map the functional topography of motor, language, and mentalizing networks during awake surgery. The empirical results highlight a robust concentration of motor and language hubs within specific cortical and subcortical clusters, while simultaneously observing a near-total resilience of the mentalizing network during the RME test. These findings should be considered exploratory and hypothesis-generating rather than definitive clinical guidance. In conclusion, our exploratory findings suggest that mapping essential motor and language hubs may indirectly support the stability of broader cognitive networks. However, given the limited sample size, these results must be interpreted with caution and serve primarily as a foundation for future, larger-scale investigations into intraoperative cognitive preservation.

## Data Availability

The data presented in this study are available on request from the corresponding authors.
